# A Phase-Intensity Surface Plasmon Resonance Biosensor for Avian Influenza A (H5N1) Detection

**DOI:** 10.3390/s17102363

**Published:** 2017-10-16

**Authors:** Chi Lok Wong, Marissa Chua, Heather Mittman, Li Xian Choo, Hann Qian Lim, Malini Olivo

**Affiliations:** Bio-Optical Imaging Group, Singapore Bioimaging Consortium, Helios #01-02, 11 Biopolis Way, Singapore 138667, Singapore; dave_wong@sbic.a-star.edu.sg (C.L.W.); marissachuasl@yahoo.com (M.C.); mittmanh@gmail.com (H.M.); andrewchoolx@gmail.com (L.X.C.); lim_hann_qian@sbic.a-star.edu.sg (H.Q.L.)

**Keywords:** SPR biosensor, surface plasmon resonance, phase-intensity detection, avian influenza A, H5N1

## Abstract

In this paper, we present a phase-intensity surface plasmon resonance (SPR) biosensor and demonstrate its use for avian influenza A (H5N1) antibody biomarker detection. The sensor probes the intensity variation produced by the steep phase response at surface plasmon excitation. The prism sensor head is fixed between a pair of polarizers with a perpendicular orientation angle and a forbidden transmission path. At SPR, a steep phase change is introduced between the p- and s-polarized light, and this rotates the polarization ellipse of the transmission beam. This allows the light at resonance to be transmitted and a corresponding intensity change to be detected. Neither time-consuming interference fringe analysis nor a phase extraction process is required. In refractive index sensing experiments, the sensor resolution was determined to be 6.3 × 10^−6^ refractive index values (RIU). The sensor has been further applied for H5N1 antibody biomarker detection, and the sensor resolution was determined to be 193.3 ng mL^−1^, compared to 1 μg mL^−1^ and 0.5 μg mL^−1^, as reported in literature for influenza antibody detection using commercial Biacore systems. It represents a 517.3% and 258.7% improvement in detection limit, respectively. With the unique features of label-free, real-time, and sensitive detection, the phase-intensity SPR biosensor has promising potential applications in influenza detection.

## 1. Introduction

Avian influenza A (H5N1) was first detected in humans in 1997 [1]. Since 2003, the WHO has recorded 844 confirmed cases, with 449 deaths [2] and a 60% mortality rate [3]. Rapid diagnosis of specific types of influenza can effectively prevent country-to-country transmission and the spread of infection during an outbreak [3]. 

Existing diagnostic technologies include viral cultures, rapid influenza detection tests (RIDT), immunofluorescence tests, and polymerase chain reaction (PCR)-based assays [4,5]. Although viral cultures provide high sensitivity, and are commonly used in many clinical settings [6], these results take 10–14 days to process. This is up to 42 times slower than immunofluorescence tests, such as direct fluorescence antibody assays (DFA), which take 2–4 h to process. However, DFAs are less sensitive (70–100%) and may be prone to photo-bleaching [6]. PCR tests are highly sensitive and specific, but require a longer processing time (about 6 h) [4,6,7], as well as trained staff needed to perform. Additionally, the costs, energy, and regulatory compliance requirements of nucleic-acid-based virus detection also limits their use in developing countries [8,9]. In recent years, several RIDT technologies have been developed, but their sensitivity is limited and false results are often reported [4,9].

The SPR sensor is a sensitive label-free technique [10,11,12,13,14,15,16,17] for the detection of bio-molecular interactions, such as antigen-antibody binding [18,19]. It also allows for a real-time response to facilitate rapid detection [20,21,22,23,24,25,26,27,28], while the system cost is low compared to surface enhanced Raman spectroscopy (SERS) [29,30] and microscopy-based optical detection [31]. Nilsson [32] recently reported inhibition-assay-based influenza virus detection. Recombinant hemagglutinin (HA) proteins of influenza A (H1N1), A (H3N2), and B were fabricated on the sensor chip surface. During detection, the mixture of influenza virus and anti-influenza serum was injected, which interacted with the surface HA proteins. Commercial Biacore^®^ T100 (Biacore, Uppsala, Sweden) was used for the measurement, and the limit of detection was measured to be 0.5 μg mL^−1^. This is an order of magnitude higher compared to single-radial immune diffusion (SRID), a commonly used method for the antibody content characterization in influenza vaccines. Additionally, Park et al. [33] proposed the detection of influenza virus via polypeptide–fusion proteins (GBP-proteins). An influenza viral surface antigen (Ala) coupled with GBP-proteins were immobilized onto the gold sensor surface. During detection, the biomolecular binding with different concentrations of anti-avian influenza antibodies was measured using a commercial Biacore 3000^TM^ SPR system. A detection limit of 1 μg mL^−1^ was reported. Zhao et al. [34] later applied side-polished SPR fiber sensor for influenza subtype H6 detection through a flow injection system. The detection limit was 5.14 × 10^5^ EID_50_/0.1ML. Currently, SPR-based avian influenza A (H5N1) detection has not been demonstrated.

Common intensity SPR sensors rely on the intensity variation produced at resonance with fixed excitation wavelength [12,35]. However, the reported sensor resolutions are limited (10^−5^ RIU) [12,36], which limits their usages in diagnosis applications. Nelson et al. [37] demonstrated the steep phase response at surface plasmon resonance and different interferometry-based phase SPR sensors were reported afterward [20,38,39]. Lam et al. [38] performed fringe analysis for dual SPR interference patterns. A detection limit of 10^−5^ RIU has been reported. Yu at al. [39] later proposed a phase SPR sensor using an interference fringe shift. In the optical design, the p- and s-polarization components were allowed to interfere with the application of a polarizing prism. This demonstrated a detection limit at 3 × 10^−5^ RIU. 

Following this, Wong and Ho et al. [18,19,21] modulated the phase of the interference images for both p- and s-polarizations in the time domain. The phase variation between the p- and s-polarized interference images at surface plasmon excitation became the sensor response. This approach shifted the formation of the interference pattern to the time domain, which is a breakthrough compared to conventional interference fringe analysis performed in the spatial domain. The sensor resolution was determined to be 9.8 × 10^−5^ RIU in a standard salt solution concentration detection. However, interference fringe-based phase SPR sensors are ultra-sensitive to environmental vibration and suffers from low sensor stability. 

In this paper, we demonstrate a phase-intensity surface plasmon resonance (SPR) biosensor. It probes the intensity change produced by the steep phase response at surface plasmon excitation. In the optical design, a prism sensor head is placed between two polarizers at a perpendicular orientation angle. At surface plasmon resonance, a steep phase variation is produced between the p- and s-polarized light, which rotates the polarization ellipse of the reflection beam from the sensing surface. The reflected light is able to be transmitted through the polarizer pair and the corresponding intensity variation is identified at the detector. This approach has demonstrated a sensor resolution of 6.3 × 10^−6^ RIU, which is an order of magnitude higher compared with interferometry-based SPR sensors. The sensor is also used for H5N1 antibody biomarker detection. To the best of our knowledge, this is the first demonstration of H5N1 antibody biomarker detection with SPR.

## 2. Materials and Methods 

### 2.1. Surface Chemistry

The gold sensing layer was immersed in 11-mercapto-undecanoic acid (0.4 mM) (Sigma-Aldrich, Singapore) for 18 h to fabricate a self-assembled monolayer. It was then washed with ethanol. A solution of N-hydroxysuccinimde (1 mM) (Thermos Scientific, Singapore) and 1-ethyl-3(2-dimethylaminoproypyl)carbodiimide (0.4 mM) (Sigma-Aldrich, Singapore) in distilled water was injected onto the modified sensor surface. One hour later, the activated sensor surface was washed with distilled water prior to protein immobilization. The functionalization process was conducted on the sensor surface before the attachment of the flow chamber. 

### 2.2. Optical Set-Up

**Figure 1:**
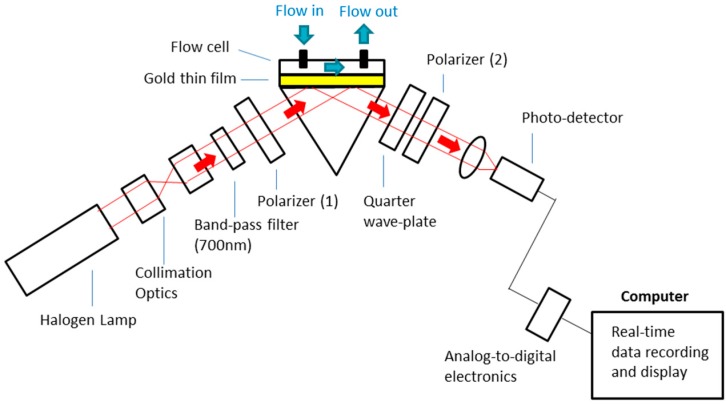
Experimental set-up of the phase-intensity surface plasmon resonance (SPR) biosensor.

Figure 1 shows the schematic of the phase-intensity SPR sensor. A halogen lamp light source was used, and a band-pass filter (FWHM—10 ± 2 nm, 700 ± 2 nm) was used to select the fixed excitation wavelength at 700 nm. In the sensor head, the reflection surface of a prism (30 × 30 mm, surface flatness—λ/4, wavelength range—400–2500 nm) was sputtered with a gold thin film (thickness ~50 nm). A polydimethylsiloxane (PDMS)-based flow cell was attached to the gold surface, which allowed different concentrations of salt solutions and bio-molecular samples to be injected onto the sensing surface for detection. In this optical design, we demonstrated phase-intensity SPR detection. The prism sensor head was placed between a pair of polarizers (wavelength range—400–700 nm, surface flatness—<1λ, transmission—25%) with perpendicular orientation, and light transmission was not allowed. At surface plasmon excitation, a steep phase shift was induced in the p-polarized light, while the s-polarized light was not affected. This generated a relative phase difference between them, and this rotated the polarization ellipse. The orientation of polarized light allowed the resultant beam to pass through the second polarizer (wavelength range—400–700 nm, surface flatness—<1λ, transmission—25%) and a corresponding intensity variation was detected with the photodetector (Si detector, wavelength range—200–1100 nm, rise time—2.3 ns, active area 0.8 mm^2^). A LabVIEW-based program has been developed for real-time signal display and recording. The sensitive phase SPR response at surface plasmon excitation provided sensitivity improvement, while measurement stability was enhanced by the non- interferometry-based phase-intensity detection approach.

## 3. Results and Discussion

**Figure 2:**
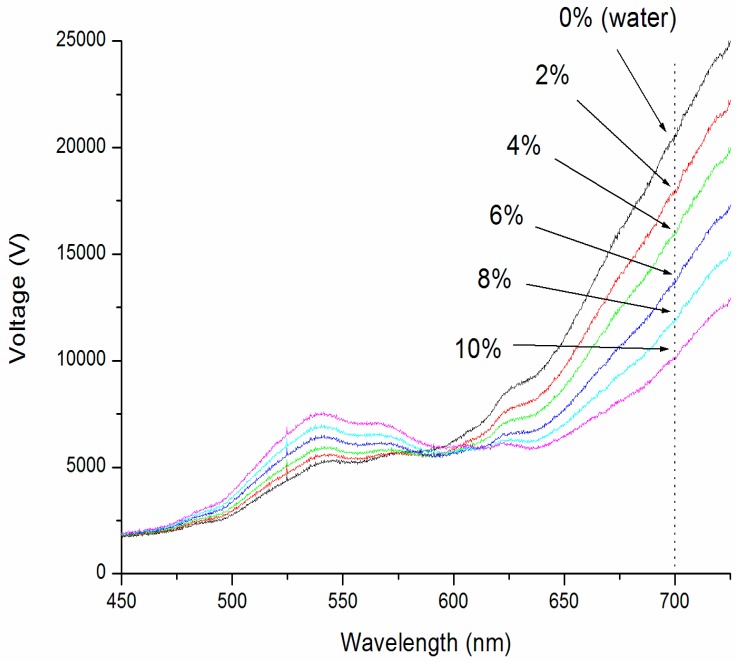
SPR spectra for salt (sodium chloride) solutions ranging from 0 to 10%, which corresponds to refractive index values between 1.3330–1.3504 RIU (refractive index unit).

To characterize the sensor resolution of the phase-intensity SPR biosensor, experimental measurements on standard refractive index samples (different concentrations of salt solutions) [10,11,12,13] have been performed. The salt solution (model 37144-00, Kanto Chemical Co., INC, Kanto, Japan) concentrations ranged from 0 to 10%, increasing by 2% increments. This corresponded to RIU ranging from 1.3330 to 1.3504 RIU. The spectral profiles for different samples were first recorded using a portable spectrometer (USB4000, Ocean Optics, Largo, FL, USA). These spectra are shown in Figure 2. It was found that the intensity difference between different refractive index samples increased with increasing wavelengths from 625 nm onward. A band-pass filter (Thorlab) was hence utilized to select the excitation wavelength at 700 nm for the phase-intensity SPR sensor. 

**Figure 3:**
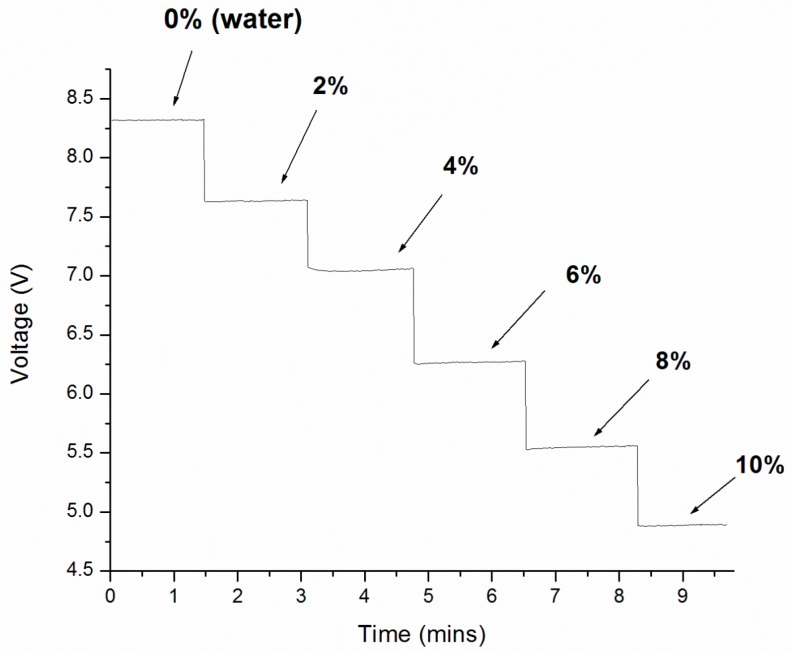
Real-time measurement results for different salt (sodium chloride) solutions ranged from 0 to 10% with the phase-intensity SPR sensor (excitation wavelength—700 nm).

Real-time measurements for different salt solutions were obtained using a home-built LabVIEW program. The results are shown in Figure 3. At the beginning of the experiment, the control sample (water, 1.3330 RIU) was injected to the sensing surface and the intensity signal was 8.3 V. The signal then decreased to 7.6 V when the 2% salt solution was injected (1.3365 RIU). The sensor signal was found to decrease with increasing solution concentration. An intensity signal of 4.9 V was recorded for 10% salt solution (1.3504 RIU). The sensor response trend matched with the spectral profile shift shown in Figure 2 at 700 nm. 

**Figure 4:**
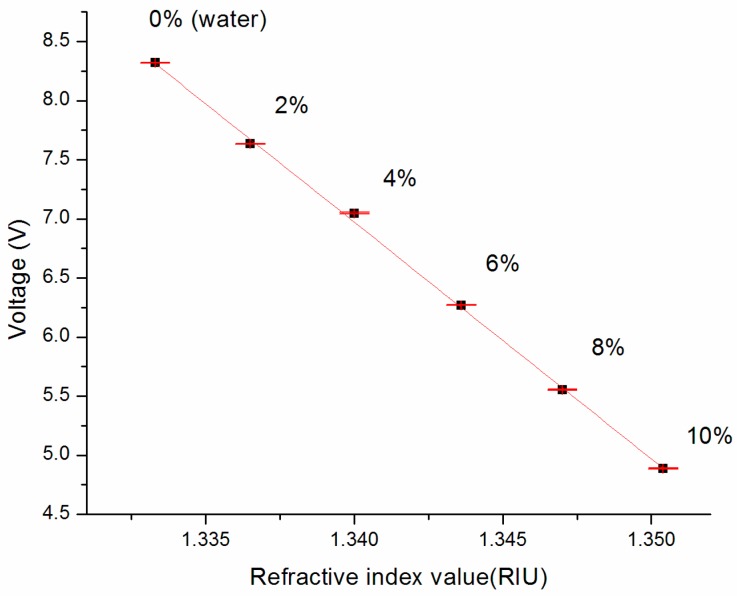
Sensor response curve. The slope is found to be 0.005 RIU/V, which provides the sensitivity of the sensor.

The average voltage responses for each sample were plotted against their refractive index in Figure 4. It gives the sensor response curve of the biosensor. According to literature, the sensitivity can be obtained using Equation (1) [10,11,19,35], and it is 0.005 RIU V^−1^.

(1) $$Sensor\;sensitivity=\;\frac{\;Refractive\;index\;changes}{Sensor\;Response}.$$

**Figure 5:**
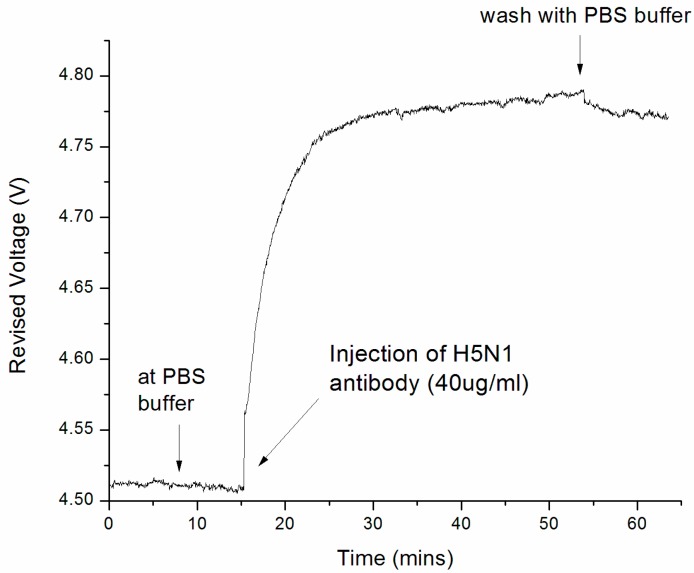
Measurement results for H5N1 antibody detection with the phase-intensity surface plasmon resonance biosensor (each data point is averaged from 10 readings). The detection resolution was found to be 193.3 ng mL^−1^, which is 517.3% [33] and 258.7% [32] greater than that reported in the literature on the influenza antibody detection, with commercial Biacore systems: Biacore 3000 [33] and Biacore T100 [32] were used in the measurement (further information is provided in Supplementary Materials, Figures S1 and S2).

Figure 5 further shows the measurement stability of the sensor. A water sample was injected onto the sensing surface and the voltage signal variations from 50 data points were recorded and the average was calculated. The standard deviation (S.D.) was 0.00127 V. In Equation (2), the sensor resolution was found to be 6.3 × 10^−6^ RIU, which is an order of magnitude higher compared with interferometry-based phase SPR sensors [21,38,39] and common intensity SPR sensors [11,36]. This value is also an improvement over the value reported by a recent intensity interrogation-based side-polished SPR fiber sensor with multiple sensing layer structure, 1.73 × 10^−4^ RIU [40]. In addition, it is comparable to the detection limit of 10^−5^–10^−6^ RIU reported by this fiber sensor in wavelength interrogation [40].

(2) Sensor resolution= Sensitivity × Measurement Stability.

In order to demonstrate the bio-sensing capability of the phase-intensity SPR sensor, it has been further applied to avian influenza (H5N1) antibody detection. The gold sensor surface was modified according to the surface chemistry procedures described in the experimental method. In the bio-sensing experiment, H5N1 virus proteins in a PBS buffer solution (0.38 mg mL^−1^) were immobilized on the modified gold surface for further detection. 

At the first stage of the experiment, the sensor surface was kept in a PBS buffer and the baseline of the bio-molecular binding curve was recorded. At 15.2 min of the experiment, H5N1 antibodies at a concentration of 40 ug mL^−1^ were injected, and the sensor response was shown in Figure 5. It was found that the sensor response increased rapidly after sample injection in the 15–25 min time frame. This is due to the bio-molecular binding between the H5N1 surface probe protein and antibody molecules. As shown in the binding curve, the binding interaction continued and became saturated in 30–50 min. This is because H5N1 antibodies have occupied a major portion of the receptor sites. At 51 min, the sensor surface was flushed with a PBS buffer to remove all non-specific bindings, and a decrease in signal of less than 5% was recorded after the washing step. This indicates that the binding signal was caused by specific binding interactions. 

The bio-sensing resolution of the sensor was obtained using Equation (3) [10,11,19,35]:

(3) $$Detection\;limit=\frac{Concentration\;of\;biomolecule\;}{Sensor\;response\;}\times \;Measurement\;stability.$$

**Figure A1:**
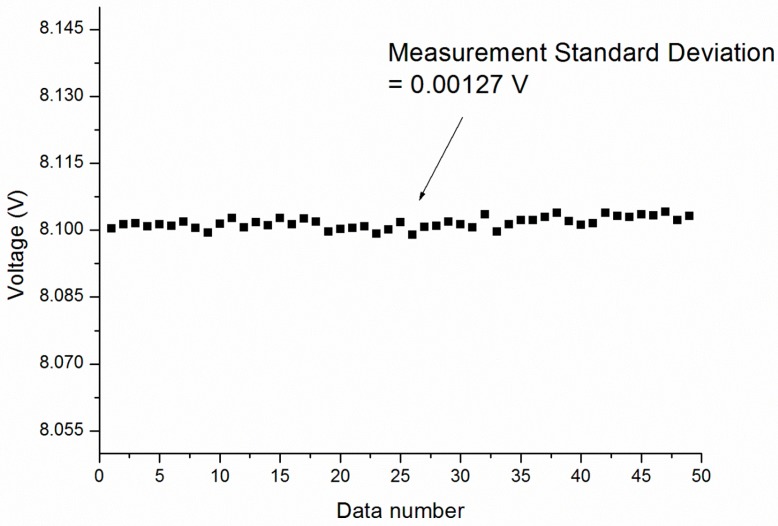
Sensor measurement stability in water sensing (1.3330 RIU). Measurement standard deviation (S.D.) was found to be 0.00127 V within 50 averaged data points (each data point is averaged from 10 readings).

With the measurement standard deviation (S.D.) indicated in Figure A1 (0.00127 V), the detection limit was determined as 193.3 ng mL^−1^ for H5N1 antibody detection, while 1 μg mL^−1^ [33] and 0.5 μg mL^−1^ [32] were reported in the literature for influenza antibody detection using commercial BIAcore systems. This represents a 517.3% and 258.7% improvement in detection limit, respectively. 

## 4. Conclusions

We have successfully demonstrated a phase-intensity SPR biosensor, which was applied for avian influenza A (H5N1) antibody biomarker detection. The sensor probes the intensity variation produced by the steep phase response at surface plasmon excitation. Neither time-consuming interference fringe analysis nor any phase extraction process is required. In refractive index sensing experiments, the sensor resolution was determined to be 6.3 × 10^−6^ RIU; one order of magnitude higher compared with interferometry-based phase SPR sensors [21,38,39] and common intensity SPR sensors [11,36]. The sensor was further applied to H5N1 antibody biomarker detection, and the detection limit was found to be 193.3 ng mL^−1^, compared to literature values of 1 μg mL^−1^ [33] and 0.5 μg mL^−1^ [32] for influenza antibody detection using commercial Biacore systems. This represents a 517.3% and 258.7% improvement in detection limits, respectively. With the unique features of label-free, real-time, and sensitive detection, the phase-intensity SPR biosensor has promising potential applications for influenza diagnosis. 

## Supplementary Materials

The following are available online at http://www.mdpi.com/1424-8220/17/10/2363/s1. Figure S1: The binding curve plotted from the direct responses for H5N1 antibody detection; Figure S2: The revised binding curve plotted from the revised sensor responses for H5N1 antibody detection.

## Appendix A

Phase-intensity-based surface plasmon resonance biosensor for avian influenza A (H5N1) antibody detection:
