# Supplementary material for: A Phase-Intensity Surface Plasmon Resonance Biosensor for Avian Influenza A (H5N1) Detection
Source: Sensors (Basel). 2017 Oct 16;17(10):2363. doi: 10.3390/s17102363 (PMC5677386; doi:10.3390/s17102363)
Supplement: Supplementary file 1 [file sensors-17-02363-s001.pdf]

sensors

MDPI

Supplementary file

The real-time binding curve plotted from the measurement results for H5N1 antibody detection with the phase-intensity surface plasmon resonance biosensor is as shown in Figure S-1. As shown in the figure, the binding curve is in negative slope (i.e. decreasing trend) for direct measurement. It matches with the sensor characteristic given by the sensor response curve in Figure 4 (voltage signal decreases with increasing refractive index). At the bio-sensing experiment, the binding of bio-molecules increases the effective refractive index on the sensor surface and decreases the voltage signal.

In order to present the data in the form of a typical binding curve. In Figure S-2 (i.e. Figure 6 in the manuscript), we calculate the revised voltage with the following equation,

$$Revised\ Voltage\ =\ Maximum\ Response\ (9V) - Sensor\ Response$$

The revised binding curve shown in Figure S-2 (Figure 6 in manuscript) shows a positive slope for increasing amount of molecular binding.

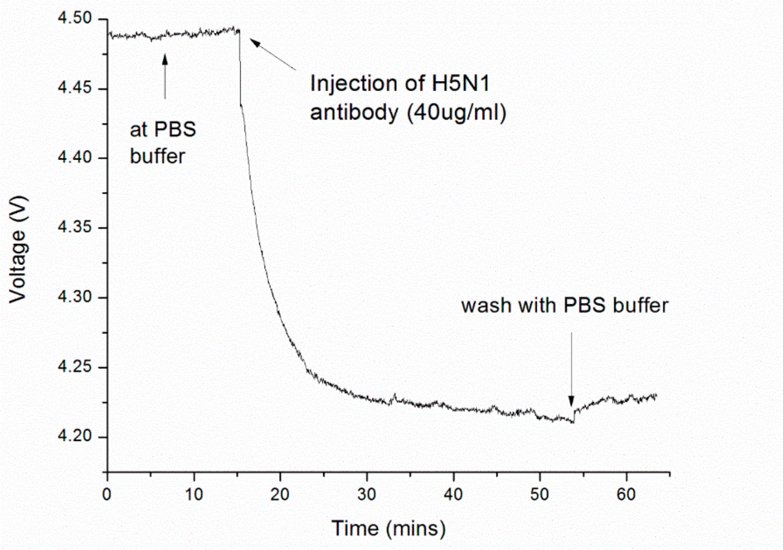

Figure S1: The binding curve plotted from the direct responses for H5N1 antibody detection.

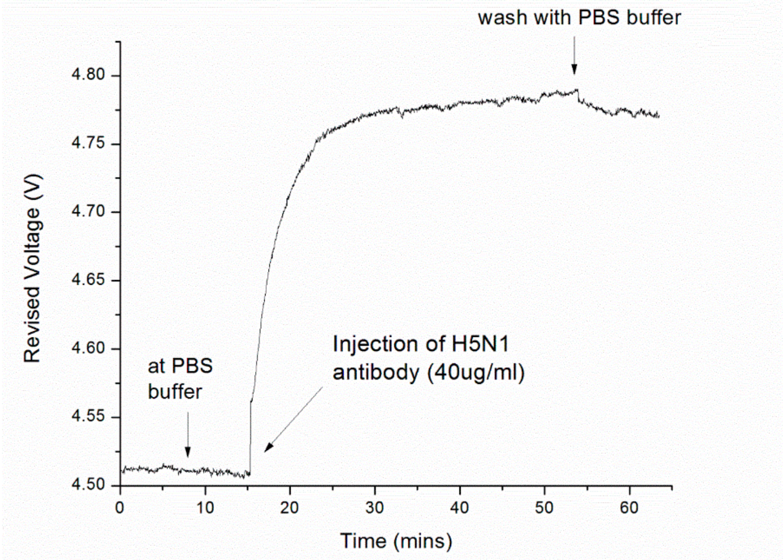

Figure S2: The revised binding curve plotted from the revised sensor responses for H5N1 antibody detection.
